# Antitumor Efficacy of α-Solanine against Pancreatic Cancer In Vitro and In Vivo

**DOI:** 10.1371/journal.pone.0087868

**Published:** 2014-02-05

**Authors:** Chongqing Lv, Hongru Kong, Guohua Dong, Lewei Liu, Kun Tong, Hongwei Sun, Bicheng Chen, Chunwu Zhang, Mengtao Zhou

**Affiliations:** 1 Department of Surgery, The First Affiliated Hospital, Wenzhou Medical University, Wenzhou, Zhejiang Province, China; 2 Zhejiang Provincial Top Key Discipline in Surgery, Wenzhou Key Laboratory of Surgery, Department of Surgery, The First Affiliated Hospital, Wenzhou Medical University, Wenzhou, Zhejaing Province, China; Taipei Medicine University, Taiwan

## Abstract

α-solanine, a steroidal glycoalkaloid in potato, was found to have proliferation-inhibiting and apoptosis-promoting effect on multiple cancer cells, such as clone, liver, melanoma cancer cells. However, the antitumor efficacy of α-solanine on pancreatic cancer has not been fully evaluated. In this study, we inquired into the anti-carcinogenic effect of α-solanine against human pancreatic cancer cells. In the present study, we investigated the anti-carcinogenic effect of α-solanine against human pancreatic cancer cells. In vitro, α-solanine inhibited proliferation of PANC-1, sw1990, MIA PaCa-2 cells in a dose-dependent manner, as well as cell migration and invasion with atoxic doses. The expression of MMP-2/9, extracellular inducer of matrix metalloproteinase (EMMPRIN), CD44, eNOS and E-cadherin were suppressed by α-solanine in PANC-1 cells. Moreover, significantly decreased vascular endothelial growth factor (VEGF) expression and tube formation of endothelial cells were discerned following α-solanine treatment. Suppressed phosphorylation of Akt, mTOR, and Stat3, and strengthen phosphorylation of β-catenin was found, along with markedly decreased tran-nuclear of NF-κB, β-catenin and TCF-1. Following the administration of α-solanine (6 µg/g for 2 weeks) in xenograft model, tumor volume and weight were decreased by 61% and 43% (p<0.05) respectively, showing decreased MMP-2/9, PCNA and VEGF expression. In conclusion, α-solanine showed beneficial effects on pancreatic cancer in vitro and in vivo, which may via suppressing the pathway proliferation, angiogenesis and metastasis.

## Introduction

Pancreatic carcinoma is one of the most aggressive and lethal cancer due to the lack of an early diagnosis, drug-resistance and poor prognosis, and as such is the fourth leading cause of cancer mortality worldwide [1], [2]. Currently, the standard treatment of pancreatic carcinoma is dependent on surgery, radiation and drugs. However, only about 25% of pancreatic cancer patients diagnosed with the resectable form attributing to the invasion of the disease [3]. Gemcitabine, a standard first–line treatment of metastatic pancreatic cancer at president, is widely used, but displays poor therapeutical effect for acquiring chemoresistance and a variety of adverse reactions [4]. Thus, seeking new effective medicines contraposing to enhance the anti–angiogenic capability and restrain metastasis is desperately needed for pancreatic cancer, targeting its most highly vascularized and invasive tumors.

In addition to the induction of cancer cell apoptosis and necrosis, inhibition of cell proliferation, infiltration and metastasis is of prime importance. Tumor metastases is a highly coordinated process which is promoted by various proteolytic enzymes degrading the extracellular matrix (ECM) and basement membrane (BM). Matrix metalloproteinases (MMPs) are believed to dominating participate in tumor cell migration, tissue invasion and metastasis [5]. MMP-2 and MMP-9 play key roles in the process of metastasis among the MMPs. Cell adhesion molecule E-cadherin, regulating cell polarity, differentiation, proliferation and migration through its intimate association to the actin cytoskeletal network [6], shows a lower expression in pancreatic cancer than in normal pancreatic tissue [7]. In addition, Wnt/β-catenin signaling cascade has been pertinent to cancer and vascular proliferation, fate specification and cell metastasis. Wnt ligand binds to its Frizzled receptor to inactivate the β-catenin destruction complex, leading to unphosphorylated β-catenin accumulation in both cytoplasm and nucleus. In the nucleus, β-catenin forms a heterodimeric complex with the TCF/LEF family of DNA binding proteins and thereby activates the transcription of Wnt target genes, compromising c-Myc, survivin, cyclinD1, and MMPs [8]. Recent studies have demonstrated that phosphatidylinositol-3-kinase(PI3K)-Akt/ mammalian target of rapamycin (mTOR) pathway is also involved in Pancreatic endocrine tumors (PETs) tumorigenesis and progression [9], [10], cell survival, cell adhesion and metastasis [11], [12]. Through crosstalk with Wnt, NF-κB and MAPK pathways, Akt/mTOR activity promotes cancer cell proliferation, inhibition of apotosis and metastasis [13], [14]. Furthermore, constitutively activated Stat proteins are found in multiple tumors [15], [16], [17], [18]. Moreover, Wnt/β-catenin and Akt/mTOR pathways regulate the expression of MMPs by transcriptional factors, such as NF-κB [19], [20]. Evidence is mounting that NF-κB plays a key role in the proliferation,apoptosis inhibition and angiogenesis of pancreatic cancer [21]. Thus, blocking Wnt/β-catenin and Akt/mTOR pathways as well as stat and NF-κB provide potential targets for cancer therapeutic strategies.

α-Solanine, a bioactive component of the main steroidal glycoalkaloids in potatoes is well studied for its impact on antitumor properties. Several reports have demonstrated that α-Solanine exhibits growth inhibition and apoptosis induction in multiple cancer cells [22], [23]. Evidence also shows α-solanine possess anti-inflammatory effects in vitro by reducing interleukin-2 and interleukin-8 productions [24]. The efficacy and the associated molecular mechanisms due to α-solanine against pancreatic cancer have not been evaluated yet. Therefore, we evaluated the efficacy of α-solanine utilizing pancreatic cancer both in vitro and in vivo in the present study.

## Materials and Methods

### Ethics Statement

In this study, animal care and experiments were conducted in accordance with an approved protocol by the Animal Experimental Ethical Inspection of Laboratory Animal Centre, Wenzhou Medical College(Permit Number:wydw2013-0001). All surgery was performed under anesthesia, and all efforts were made to minimize suffering.

### 1. Cell Line and Reagents

α-solanine, dimethyl sulfoxide (DMSO) were purchased from Sigma-Aldrich(St. Louis, MO, U.S.A.). Protein assay kit was obtained from Bio-Rad Labs (Hercules, CA, U.S.A.). Dulbecco’s modified Eagle’s medium (DMEM) and was purchased from Gibco/BRL (Gaithersburg, MD, U.S.A.). Matrigel was from BD Biosciences (Bedford, MA, U.S.A.). Total RNA extraction kit and polymerase chain reaction (PCR) kit were from Viogene (Sunnyvale, CA, U.S.A.). Antibodies against MMP-2, MMP-9, E-cadherin, TCF-1, STAT3 and phosphorylated STAT3 were purchased from Cell Signaling Technology (Danvers,MA, U.S.A.). Antibodies against β-actin, VEGF, PCNA, β-catenin, Akt, mTOR, phosphorylated proteins were purchased from Abcam(Cambridge, MA, USA). PANC-1, sw1990, MIA PaCa-2 and Human umbilical vein endothelial cell(HUVEC) were obtained from ATCC (Manassas, VA, USA). Pancreatic cancer cell lines are maintained in DMEM with 10% FBS and incubated in a 5% CO_2_ humidified incubator at 37°C, HUVECs were cultured in M199 medium with 10% FBS. α-Solanine was melted in DMSO and diluted with culture medium (the final concentration of DMSO was less than 0.1%).

### 2. Cell Viability Assay and Colony Assay

Seeding 20,000 cells of each cancer cell line in each well of 96-well plate and replacing the culture medium containing various concentrations of α-solanine(3,6,9,12 µg/µl) after 24 h. Continuing to develop for 24 and 48 h, then the medium was replaced with the fresh medium inclusion of 10% CCK8 (Dojindo Laboratories, Kumamoto, Japan). After 1–4 h, the supernatants were measured spectrophotometrically at 450 nm.

Anchorage-independent cell growth is evaluated by performing colony assay (GENMED SCIENTIFECS INC, USA) according to the manufacturer’s instructions. In brief, 1.5 ml Base Agar Matrix Layer is dispensed into each well of a 12-well plate (samples were assayed in triplicate). Plate was chilled at room temperature until solid. Then 1.5 ml growth agar layer consisting of 2500 cells was added into each well. Plate was chilled at room temperature again until the growth layer congealed. A further 500 µl culture media containing various concentrations of α-solanine was added on top of the growth layer. Incubate the cells at 37°C and 5% CO_2_ for 2 weeks and total colonies were counted.

### 3. Cell Migration and Invasion Assays

Cell migration was studied by performing wound healing assays. 10,000 cells of each pancreatic cancer cell line were plated in high 35-mm μ-dishes with culture inserts (Ibidi, Martinsried, Germany). When cells grew to confluence, inserts were then removed with sterile forceps to create a wound field of approximately 500 µm. After removing the cellular debris with PBS, cells were exposed to various concentrations of α-solanine for 24 h. Cell migration were perceived by inverted microscope and photographed (100×magnification). The wound area was scaled by Image Pro Plus. The wound closure percent was calculated by the equation: Wound closure % = [1-(wound area after 24 h/wound area after 0 h)×100%.

Cell invasion assays were implemented using 6.5 mm transwell chambers equipped with 8.0 µm pore-size polycarbonate membranes. In these assays, the upper champers were first coated with 50 µl of Matrigel at a 1∶5 dilution, and incubated at 37°C for 2 h. After treated with α-solanine for 24 h, serum-free cells (10,000 cells/well) suspension medium of each pancreatic cancer cell line were loaded onto the top chamber of the transwell. The lower chambers were filled with 500 µl DMEM supplemented with 10% FBS. After incubation at 37°C for 6 h, non-invasive cells were physically scraped from the membrane with the cotton swabs. The invasive cells were stained with DAPI for 5 minutes and observed with a fluorescent microscope (Leica Microsystems, Wetzlar, Germany). Fluorescent cells were counted using Image Pro Plus.

### 4. In Vitro Angiogenesis Assay

Analysis of capillary formation was performed using tube formation assays (Ibidi, Martinsried, Germany) according to the manufacturer’s instructions. Briefly, a 15-well μ-Slides was coated with 10 µl of Matrigel which was allowed to solidify at 37°C. To evaluate the effect of α-solanine, PANC-1 cells were treated with various concentrations of α-solanine for 24 h, then the conditioned medium were collected. HUVEC were put into the μ-Slide well and incubated with conditioned medium of PANC-1 cells for 6 h. The generation of cellular networks were photographed and quantitatively evaluated by Image Pro Plus.

### 5. Quantitative Real-time Polymerase Chain Reaction

Total RNA was extracted from PANC-1 with TRIzol Reagent (Ambion, Carlsbad, California, USA) following the manufacturer’s instructions. Total RNA (1 µg) of each sample was subjected to oligo-dT-primed RT with ReverTra Ace qPCR RT kit (Toyobo, Osaka, Japan). Realtime polymerase chain reaction (PCR) was performed for a quantitative analysis of MMP-2, MMP-9, VEGF, EMMPRIN, E-cadherin and CD44 mRNA expression using SYBR Green real-time PCR Master Mix (Toyobo) on a 7500 Real Time PCR System (Applied Biosystems, Foster City, CA, USA). PCR conditions were as follows: 95°C for 1 min, 40 cycles at 95°C for 15 s and 60°C for 60 s. The primer sequences for GAPDH, MMP-2, MMP-9, VEGF, ENOS, EMMPRIN, E-cadherin and CD44 were synthesized from Invitrogen and listed in Table 1. Analysis of quantitative realtime PCR data was performed on ΔCt values.

**Table 1:** Prime pairs used in Realtime PCR.

Genes	Primers(5′–3′)		Product length(bp)
MMP-2	Forward	AATGCCATCCCCGATAACC	120
	Reverse	GCTCAGCAGCCTAGCCAGTC	
MMP-9	Forward	GGGGGAAGATGCTGCTGTT	140
	Reverse	AGCGGTCCTGGCAGAAATAG	
EMMPRIN	Forward	ACAAAGATGGTCACGGTCTGCC	242
	Reverse	ACCAAGAAGCTGAGCGAGTGTC	
CD44	Forward	ATGGACAAGTTTTGGTGGCA	184
	Reverse	TGGGCAAGGTGCTATTGAAAG	
ENOS	Forward	AAGAGGTGGAAGCCGAGGT	207
	Reverse	TGGTGGCATACTTGATGTGGT	
E-cadherin	Forward	GAGAAGAGGACCAGGACTTTGACT	204
	Reverse	AATCATAAGGCGGGGCTGT	
GAPDH	Forward	GGGTGTGAACCATGAGAAGTATG	145
	Reverse	GATGGCATGGACTGTGGTCAT	

### 6. Protein Extraction and Western Blot Analysis

PANC-1 cells were lysed in radioimmune precipitation assay (RIPA) buffer containing protease and phosphatase inhibitor (Roche, Basel, Switzerland). Nuclear and cytoplasmic proteins were extracted with the Nuclear and Cytoplasmic Extraction Reagents (Beyotime, Jiangsu, China) according to the manufacturer’s instructions. Proteins were fractionated by sodium dodecyl sulfate-polyacrylamide gels electrophoresis and transferred to PVDF membrane (Solarbio). Membranes blocked with 5% nonfat dry milk were incubated with relevant antibodies overnight at 4°C. Then the membranes were washed three times with TBST and incubated with secondary antibodies for 1 h at room temperature. After washing three times with TBST, target bands were detected using ECL (Advansta, Menlo Park, California, USA) and exposed on a film. The density of protein bands were measured by Quantity One.

### 7. Analysis of MMP-2 and MMP-9 Activities by Gelatin Zymography

The activities of MMP-2 and MMP-9 were assayed by gelatin zymography. PANC-1 cells were incubated with serum-free medium with various concentrations of α-solanine for 24 h. The conditioned medium was then collected and concentrated. Each sample (20 µg) was mixed with loading buffer and subjected into 10% SDS-polyacrylamide gel containing 0.1% gelatin. Electrophoresis was performed at 100 V for 1.5 h at 4. Gels were then washed with washing buffer (2.5% Triton X-100, 50 mmol/L Tris –HCl, 5 mmol/L CaCl_2_, pH7. 6), followed by incubation at 37°C in reaction buffer (50 mmol/L Tris - HCl, 5 mmol/CaCl2, 0. 02% Brij-35, pH7.6). After 42 h, the gels were stained with Comassie blue (0.05% Comassie blue, 30% methanol, 10% acetic acid) for 3 h and destained with destaining solution (20% methanol, 10% acetic acid, 70% ddH2O) until the clear bands were visualized.

### 8. Animals and Tumors

Athymic (nu/nu) male nude mice were obtained from the Shanghai Lab. Animal Research Center(Shanghai, China) and housed under standard laboratory conditions (pathogenfree conditions with a 12 h light/12 h dark schedule). To produce tumor animals, 5×10^6^ PANC-1 cells were subcutaneously injected into the flanks of four-week-old athymic nu/nu male mice. When the tumors were measurable, the mice were divided into two groups(6/group) randomly. Animal care and experiments were conducted in accordance with an approved protocol by Animal Experimental Ethical Inspection of Laboratory Animal Centre, Wenzhou Medical College.

### 9. Treatment Method and the Tumor Xenografts Study

The control group, in which 1 µl/g(weight of mice) DMSO was injected into the abdominal cavity of each mouse; the solanine group,in which 1 µl/g(weight of mice) solanine was injected in the same way. The concentration of solanine is 5 µg/µl. Solanine had a remarkable safety at the dose of 5 µg/g(weight of mice), and there were no clinical and histological differences between the treatment and the control groups [25]. Each group was treated with drug 1 time per day for two weeks. The body weight of mice and their tumor sizes were measured every day. Mice were sacrificed 2 weeks after drug administration and the tumors were carefully separated and weighed. The tumor volume was calculated by the formula: 0.5236 L1(L2)^2^, where L1 is long diameter, and L2 is short diameter. Part of the tumor was used for Western blot and stored in 4% paraformaldehyde for immunohistochemistry, and the rest was frozen in liquid nitrogen.

### 10. Western Blot for MMP-2 and MMP-9 and Immunohistochemical Staining for PCNA and VEGF

The tumor xenograft protein extraction procedure and treating processes is as same as that mentioned above. Contiguous 4-µm sections were cut from paraffin embedded tumor tissues for immunohistochemistry. Sections were blocked with goat serum and immunostained after deparaffinization and rehydration. Sections were then incubated with a 1/200 dilution of primary antibody against PCNA or VEGF for overnight at 4°C, followed by incubating with secondary antibodies for 60 min at room temperature. The slides were developed with diaminobenzidine and counterstained with hematoxylin. The proliferation index (per 400×microscopic field) was determined as number of PCNA-positive cells/total number of cells×100. The integrated optical density (IOD) was analyzed for VEGF quantification. 6 fields were selected randomly from each section. The average IOD levels between two groups were then compared. Image-Pro Plus 6.0 software was used for analysis.

### 11. Statistical Analysis

All analysis were carried out by SPSS17.0 software. Data shown are representative images or expressed as means ± S.E.M. of each group. The difference among groups of cells was analyzed using ANOVA, and Independent Sample T test was used for analysis between groups in vivo. P<0.05 was considered statistically significant.

## Results

### 1. α-solanine Affect Cell Viability and Inhibits Colony Formation

**Figure 1:**
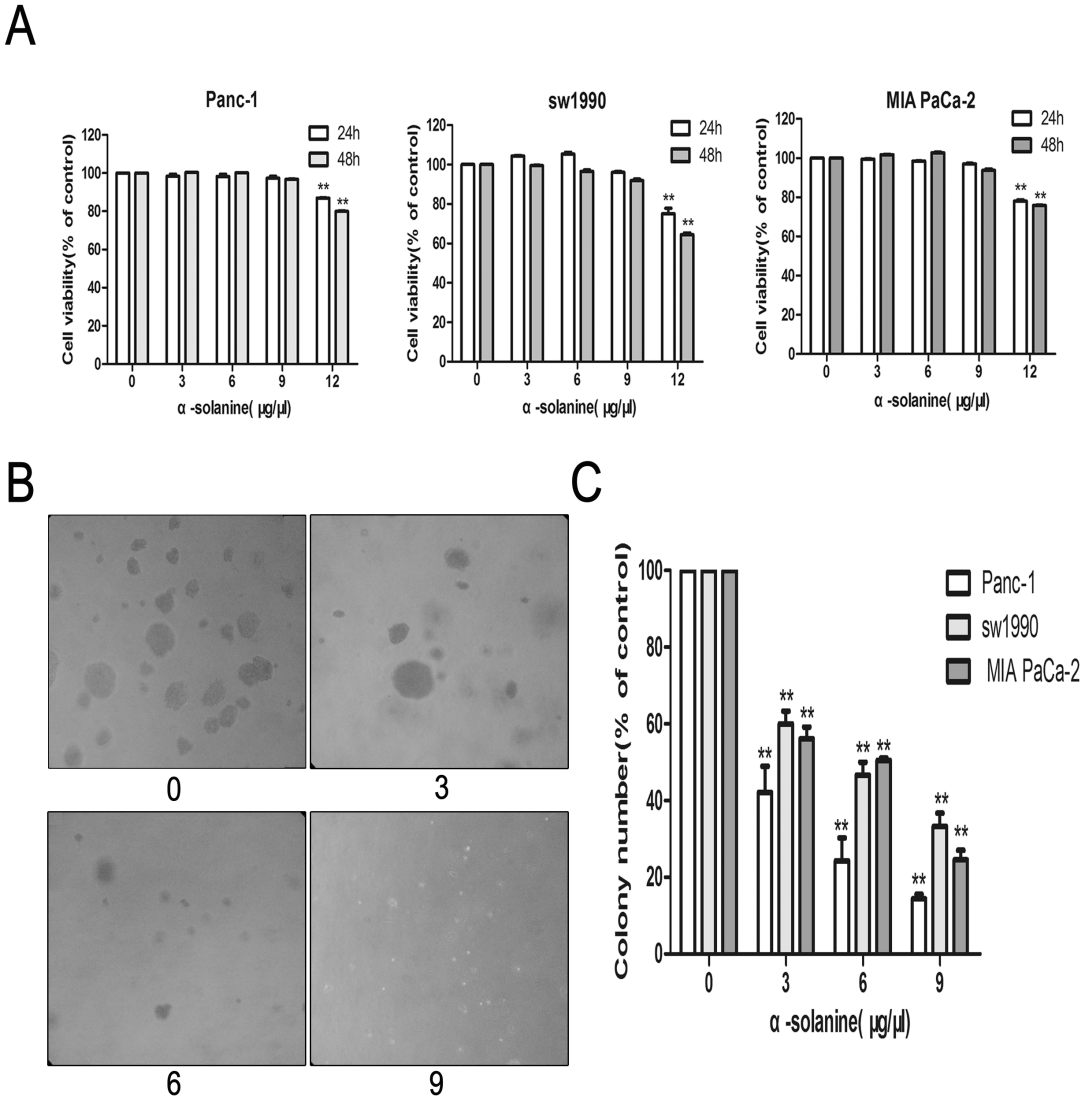
Cell Viability Assay and Colony Assay. (A) Cells of each cancer cell line were treated with various concentrations of α-solanine for 24 h and 48 h. (B) Photos of anchorage-independent cell growth of PANC-1(100×magnification). (C) The colony number were quantified and data were calculated from three independent experiments. Each bar represents the means ± S.E.M. (n = 3). **p<0.01, compared with the control.

We found that the treatment of α-solanine(3,6,9 µg/µl) for 24 h or 48 h did not change the viability of PANC-1, sw1990 and MIA PaCa-2 cells significantly(Fig. 1A). Cell viability was declined significantly after treatment of α-solanine at 12 µg/µl. The results revealed that cytotoxicity of cells of each cell line were not caused by α-solanine at 3, 6, 9 µg/µl for 24 h and 48 h. We used non-toxic doses of α-solanine for experiments in vitro.

To elucidate the functions of α-solanine in the anchorage-independent growth of pancreatic cancer cells, we utilized a soft agar assay. Cells without α-solanine treated formed more and bigger colonies than α-solanine treated cells (Fig. 1B,C), indicating that anchorage-independent growth of pancreatic cancer cells was inhibited by α-solanine in a dose-dependent manner.

### 2. α-Solanine Inhibits Cell Migration and Invasion

**Figure 2:**
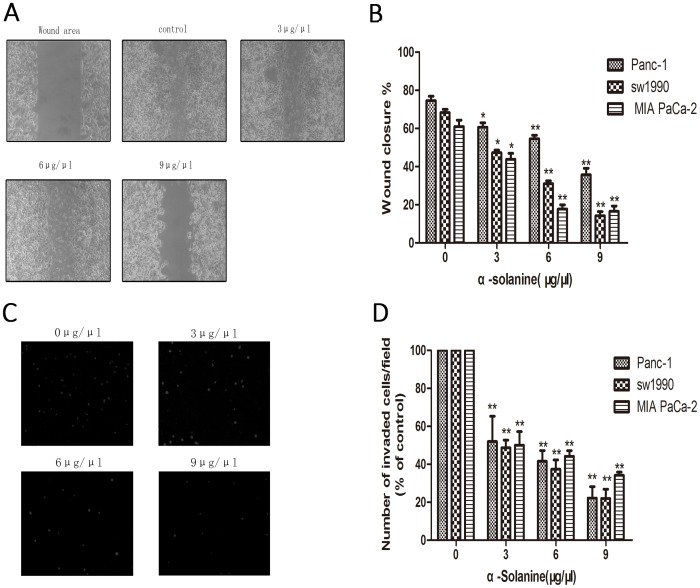
Cell Migration and Invasion Assays. Cells were treated with various concentrations of α-solanine for 24 h. (A) Cells were photographed(100×magnification). (B) The wound area were quantified in four fields in each treatment, and data were calculated from three independent experiments.(C) Cells were photographed(100×maginfication). (D) The invaded cells were quantified by counting fo DAPI-stained cells. Each bar represents the means ± S.E.M. (n = 3).*p<0.05, **p<0.01, compared with the control.

Wound healing assay and transwell invasion assay was performed to observe the effect of α-solanine on cell maigratoin and invasion. The results showed that α-solanine suppressed migration and invasion of pancreatic cancer cells in a dose-dependent manner (Fig. 2).

### 3. α-Solanine Inhibited Tube Formation of PANC-1 Induced ECs and Expression of VEGF

**Figure 3:**
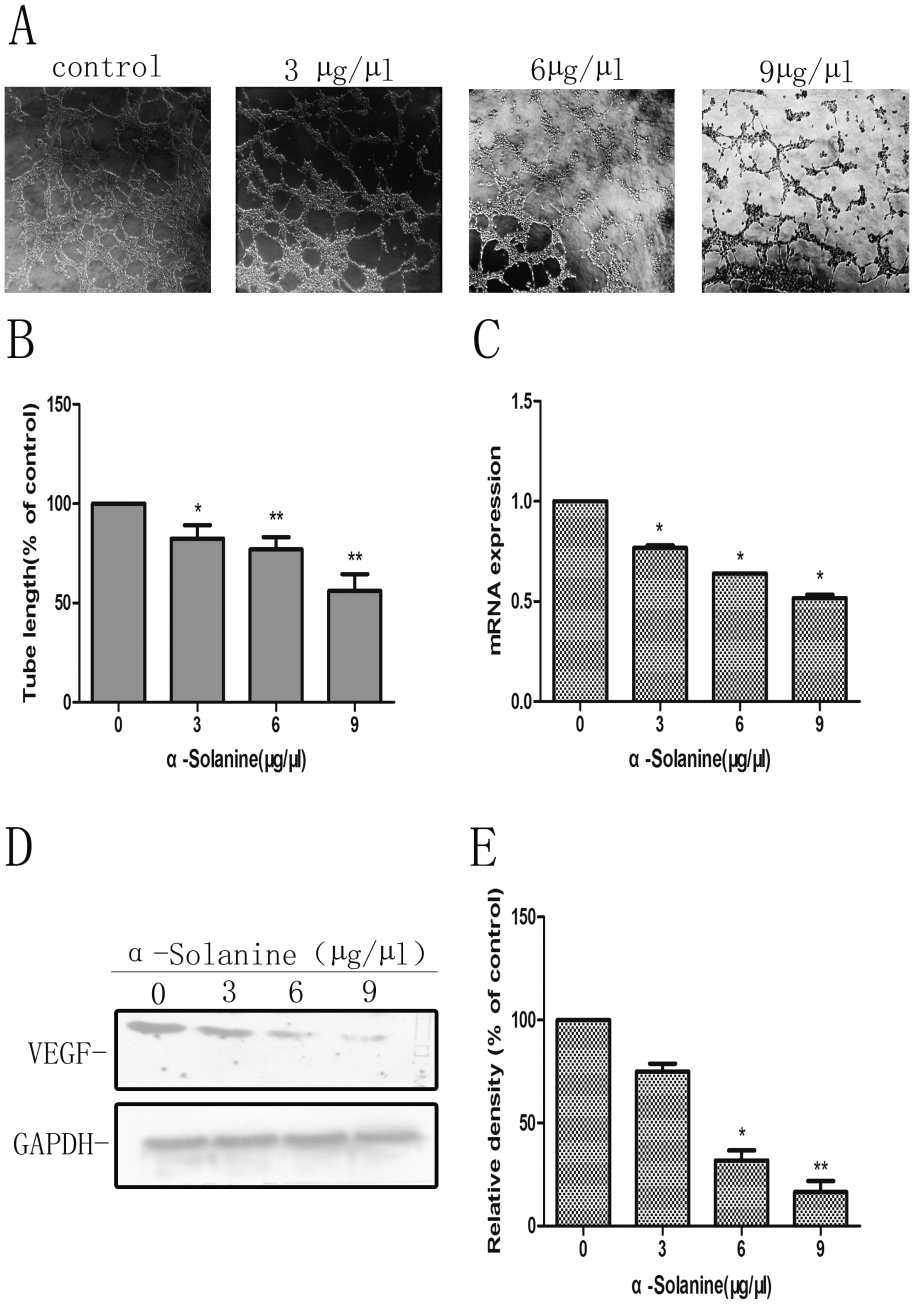
In Vitro Angiogenesis Assay. (A) Representative micrographs (100×) of tube after treatment of conditioned medium for 6 h. HUVECS were plated onto the Matrigel –precoated wells and cultured in conditioned medium from PANC-1 cells treated with for 24 h. (B) The tube lengths were measured by Image-ProPlus 6.0. (C) The mRNA expression of VEGF was presented as means ± S.E.M. of three independent experiments.(D) Western blot analysis of the expression of VEGF. GAPDH was used as a loading control. (E) Quantification of the western blot result was performed by calculating the ratio of the value to the control group. *p<0.05, **p<0.01, compared with the control.

We found that conditioned media of PANC-1 cells induced tube formation of HUVEC, while conditioned media from PANC-1 treated by α-Solanine suppressed tube formation of HUVEC in a dose–dependent manner (Figs. 3A, 3B). VEGF, as a angiogenic factor, promotes angiogenesis. Our results also showed that α-Solanine(3, 6 and 9 µg/µl) decreased markedly mRNA and protein expression of VEGF in PANC-1 cells in a dose–dependent manner (Figs. 3C, 3D and 3E). Data revealed that α-Solanine inhibits angiogenesis in by restraining VEGF expression.

### 4. α-Solanine Exerts Expression of Metastasis Associated Molecule

**Figure 4:**
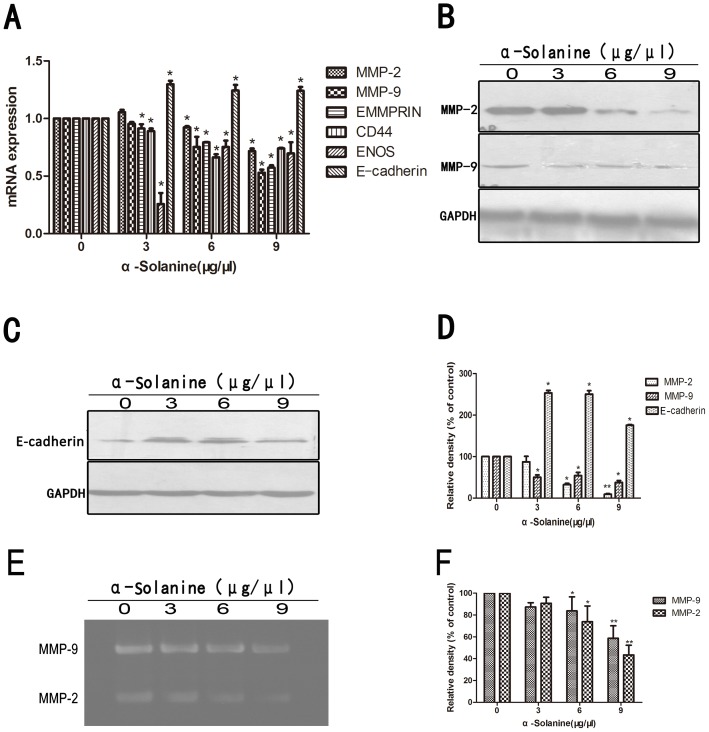
Effect of α-Solanine on expression of metastasis associated molecule. (A) The mRNA expression of MMP-2/9, EMMPRIN, CD44, ENOS and Ecadherin was presented as means ± S.E.M. of three independent experiments.(B) The expressions of MMP-2 and MMP-9 protein were analyzed by Western blot.(C) The expressions of Ecadherin protein was performed by Western blot.(D) Quantification of the western blot results were performed by calculating the ratio of the value to the control group.(E) The activity of MMP-2 and MMP-9 were analyzed by Gelatin Zymography. (F) Quantification of the Gelatin Zymography results were performed by calculating the ratio of the value to the control group. *p<0.05, **p<0.01, compared with the control. GAPDH was used as a loading control.

Expression of genes associated with cancer progression and metastasis was performed by quantitative real time PCR(Figs. 4A). The activation of MMPs is a crucial step for ECM degradation, which induces cell invasion. The results showed that α-Solanine suppressed the mRNA expression of MMP-2, MMP-9, ENOS, EMMPRIN and CD44 in a dose dependent manner. Protein expression of MMP-2 and MMP-9 were also suppressed in a dose dependent manner (Figs. 4B, 4D). Gelatin zymography assay also showed that MMP-9 and MMP-2 activities were markedly reduced by 6 and 9 µg/µl α-Solanine (Figs. 4E, 4F). Moreover, α-Solanine unregulated the expression of E-cadherin(Figs. 4C,4D), which can increase the adhesion between the cells. Thus, α-Solanine could inhibit metastasis by affecting the proteolytic activation and adhesive capacity.

### 5. α-Solanine Regulated Associated Signaling Proteins

**Figure 5:**
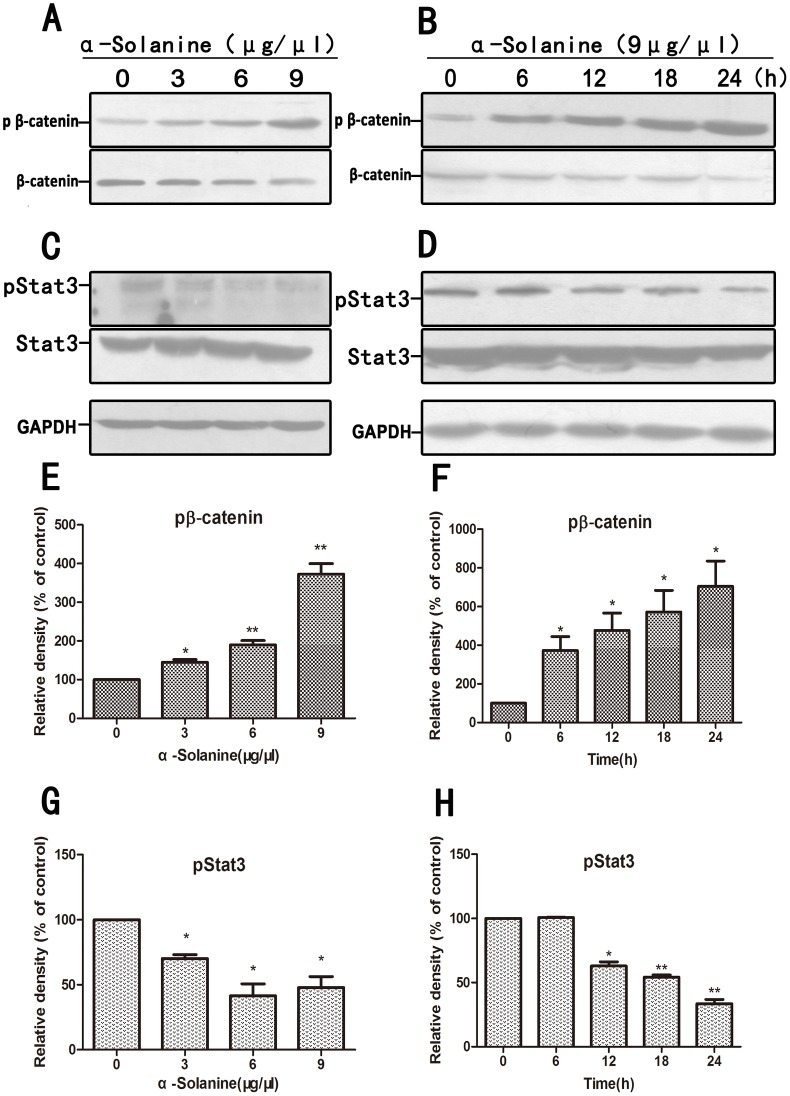
Effect of α-solanine on phosphorylation of β-catenin and Stat3. PANC-1 cells were treated with various doses of α-solanine for 24 h, or 9 µg/µl of α-solanine for 6,12,18,24 h. The phosphorylation level of β-catenin(A,B), Stat3(C,D) were determined by Western blot. GAPDH was used as a loading control.(E,F,G,H) Quantification of the western blot results were performed by calculating the ratio of the value to the control group. *p<0.05, **p<0.01, compared with the control.

**Figure 6:**
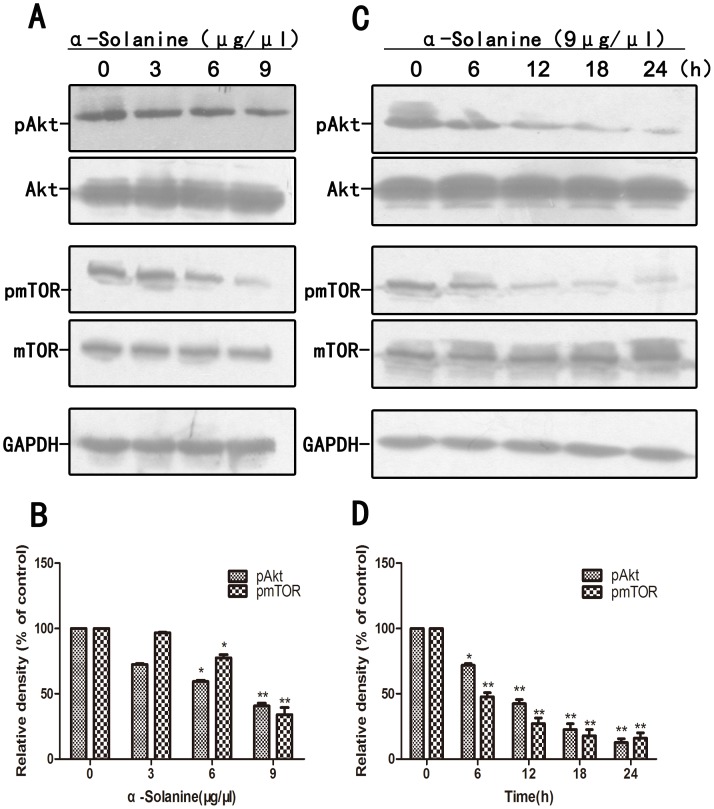
Effect of α-solanine on phosphorylation of Akt and mTOR. PANC-1 cells were treated with various doses of α-solanine for 24 h, or 9 µg/µl of α-solanine for 6,12,18,24 h. (A,C) The phosphorylation level of Akt and mTOR were determined by Western blot. GAPDH was used as a loading control. (B,D) Quantification of the western blot results were performed by calculating the ratio of the value to the control group. *p<0.05, **p<0.01, compared with the control.

**Figure 7:**
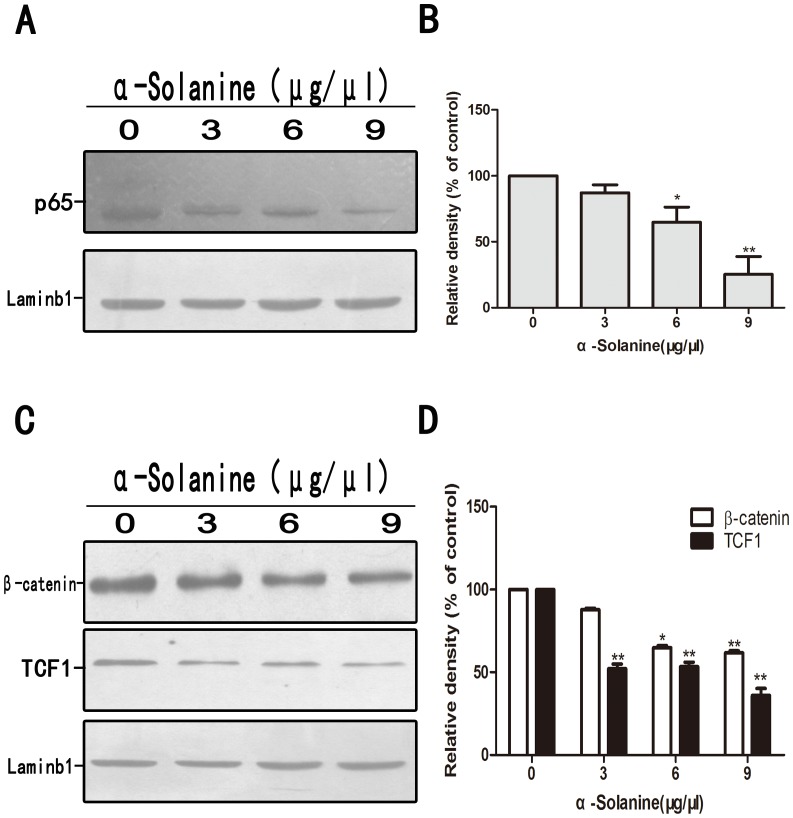
Effect of α-solanine on the expression of NF-κB/p65, β-catenin and TCF-1 in nucleus of PANC-1 cells. PANC-1 cells were treated with various doses of α-solanine for 24 h. (A,C) The level of NF-κB/p65, β-catenin and TCF-1 in nucleus were determined by Western blot. Laminb1 was used as a nuclear protein loading control. (B,D) Quantification of the densitometric results were performed by calculating the ratio of the value to the control group. *p<0.05, **p<0.01, compared with the control.

Research indicated that Wnt/β-catenin, Akt/mTOR and JAK/STAT pathways are involved in the expression of MMPs and inducing metastasis. α-Solanine induced the expression of phosphorylation of β-catenin and inhibited the phosphorylation of STAT3 in a dose and time dependent manner(Figs. 5). Data also showed that α-Solanine reduced the phosphorylation of Akt and mTOR in a dose and time dependent manner (Figs. 6). In addition, α-Solanine down-regulated the level of β-catenin and TCF-1 in nucleus (Figs. 7). Thus, α-Solanine could suppress cell invasion and MMP-2/9 expression partly via inhibiting Wnt/β-catenin, Akt/mTOR and JAK/STAT pathways.

### 6. α-Solanine Decreased the Expression of NF-κB/p65 in Nucleus of PANC-1 Cells

The relative nuclear level of NF-κB/p65 in PANC-1 cells was determined using the Western blot assay. We found that treatment with α-Solanine significantly decreasd the expression of NF-κB/p65 in PANC-1 cells (figs. 7A, 7B). Constitutive activation of NF-κB can promote cell proliferation, inhibit cell apoptosis, and regulate the expression of genes associated with angiogenesis. Thus α-Solanine could enhance the inhibition of cell growth and proliferation and promote pancreatic cancer cell apoptosis by inhibiting the expression of NF-κB.

### 7. α-Solanine Suppresses in vivo Growth of Pancreatic Cancer Cell PANC-1 Tumor Xenografts

**Figure 8:**
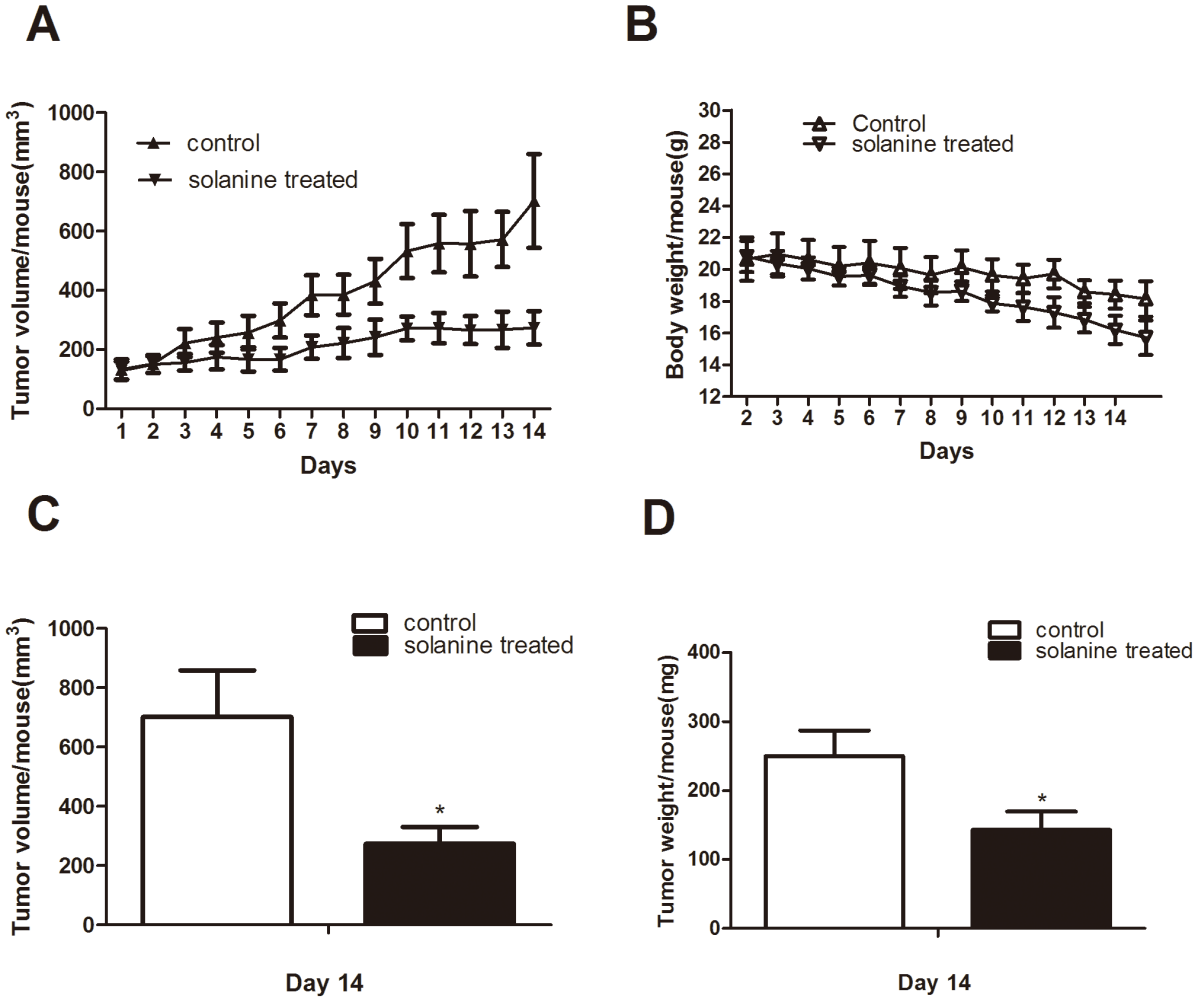
Effect of α-solanine on the PANC-1 tumor xenograft growth and body weight of athymic nude mice. PANC-1 cells were subcutaneously injected into the flanks of nude mice. When the tumors were measurable, mice were given DMSO or α-solanine for 2 weeks. (A) Tumor volume/mouse as a function of time.(B) The mouse of each group was monitored for body weight once a day. Mean body weight/mouse as a function of time.(C) Tumor volume/mouse and (D) tumor weight/mouse at the end of the study were analyzed. *p<0.05, compared with the control.

Administration of solanine to nude mice inhibited xenograft growth of PANC-1 tumor (Figs. 8). At the end of 14 days of the study in PANC-1 xenograft, solanine decreased tumor volume/mouse from 701.97±157.86 mm^3^ in control group to 273.54±57.27 mm^3^, corresponding to a 61% (P<0.05) reduction in tumor volume. Similarly,tumor weight in solanine treated group was also decreased from 250±37.42 mg in control group to 143.33±26.29 mg, corresponding to a 43% (p<0.05) reduction in tumor weight. We observed there was a little drop in body weight both in control group and in solanine treated group. The reason of that was not clear yet.

### 8. α-Solanine Suppresses Metastasis, Cell Proliferation and Angiogenesis in Xenografts Model

**Figure 9:**
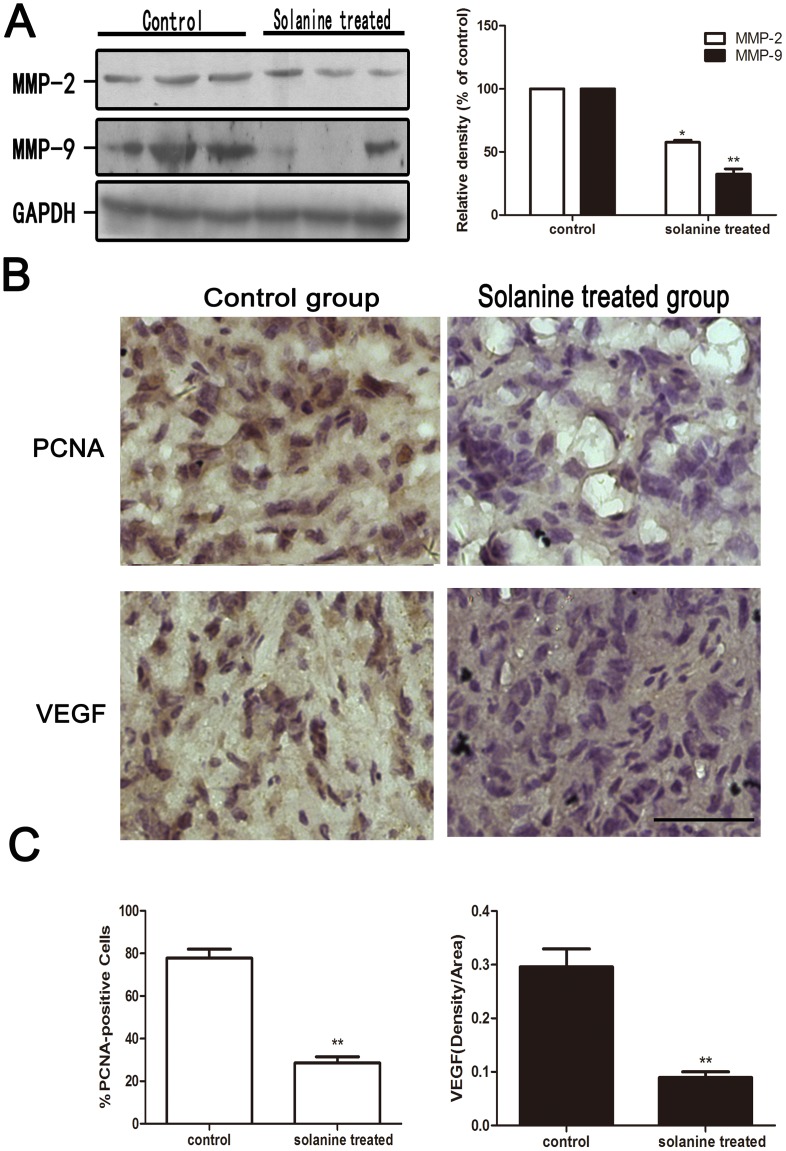
Effect of α-solanine on the expression of MMP-2, MMP-9, PCNA and VEGF in PANC-1 tumor xenograft. When tumor xenografts were seperated, parts of tumor were used for Western blot and Immunohistochemistry. (A) The expressions of MMP-2 and MMP-9 protein were analyzede by Western blot. (B,C) Tumor tissus were innunohistochemically analyzed for PCNA-positive cells and VEGF staining density. Representative photographs of IHC staining of PCNA and VEGF are shown at 400×magnifications. Scale bar: 50 µm. The data are presented as the mean ± SE. *p<0.05, **p<0.01, compared with the control.

MMP-2 and MMP-9 play key roles in the process of metastasis among the MMPs. We examined pancreatic tumor xenograft by Western blot and found α-Solanine can significantly decrease the expression of MMP-2 and MMP-9(Figs. 9A, 9B). Proliferation and angiogenesis are the two extensively used biomarkers, which have been employed for measuring the aggressiveness of solid tumors. Hence, tumors were analyzed for anti-proliferation and anti-angiogenesis of α-Solanine by PCNA and VEGF staining through immunohistochemical methods. The PCNA staining and VEGF staining of tumors showed poor immunoreactivity in solanine treated group as compared to control group (Figs. 9B,9C). The quantification showed 78±4% PCNA-positive cells in control group versus 29±3% in solanine treated group accounting for 63% decrease(p<0.01), and the average IOD of VEGF staining performed 70% decrease(p<0.01) in solanine treated group compared with control group. This study confirmed in vivo antitumor mechanism of solanine efficacy against PANC-1 tumor growth.

## Discussion

α-Solanine is a kind of steroid alkaloids which produced by the plants of the sloanaceae family. High concentration of α-Solanine can generate cytotoxic effects which induce rapid damage of plasma membrane causing the lethal disorder of metabolism [26]. Several studies show that α-Solanine inhibits pre-implantation embryo development [27], [28], normal human liver cells [22]. Moreover, α-Solanine has been shown to reduce cytokine and nitric oxide productions in Con A-induced Jurkat cells and LPS-stimulated Raw macrophages [24], inhibit tumor necrosis factor-α(TNF-α) and interleukin (IL)-6 in mouse peritoneal macrophages due to the suppression of p38,JNK and ERK1/2 [29]. Recent studies have demonstrated that α-Solanine performs anti-tumor efficacy, such as restraining the proliferation of cancer cell lines and inducing apoptosis and decrease of mutation of p53 of gastric cancer cell lines [22], [23], [30]. In the present study, we used non-toxic concentration of α-Solanine(3, 6 and 9 µg/µl) and found that α-Solanine inhibits metastasis in vitro, such as invasion, migration and angiogenesis, indicating that the inhibitory effect of α-Solanine on metastasis was not by its cytotoxic function. We first evaluated the efficacy of α-Solanine in vivo and found α-Solanine can inhibit proliferation, angiogenesis and metastasis in tumor xenograft in athymic nude mice.

Cancer metastasis is a multifaceted process which genetically unstable cancer cells develop adaptation and ecesis to a tissue microenvironment that is distant from the primary tumor [31], involving loss of cellular adhesion, increased migration and invasion, circulation through the vascular/lymphatic systems and germination of colonial tumors at distant sites [32], [33]. Degradation of ECM and BM by proteolytic enzymes and pursuant invasion are indispensable for metastasis [34]. MMPs are the important proteolytic enzymes which degrade the ECM and BM. Among them, MMP-2 and MMP-9 are highly expressed in pancreatic cancer [35]. Inhibition of pancreatic cancer cell metastasis mediated by downregulating the expression of MMP-2 and MMP-9 [36], [37]. EMMPRIN, stimulating peritumoral fibroblasts to produce MMPs, performed with a particular high expression in pancreatic cancer cell [38]. Pancreatic cancer cell invasion and metastasis could be suppressed through anti-EMMPRIN therapy [39]. CD44, a transmembrane glycoprotein with binding domains for hyaluronic acid which is crucial component of ECM, is highly expressed in pancreatic cancer [40]. Moreover, E-cadherin is a key molecule involved in conditioning cell-cell adhesion. The loss of E-cadherin provides cancer cells with an invasive ability. In this study, we demonstrated that α-Solanine significantly suppressed migration and invasion of pancreatic cancer cell line PANC-1, sw1990 and MIA PaCa-2 cells at a dose–dependent manner. The protein expression level of MMP-2 and MMP-9 were subdued in vitro by α-Solanine, while inducing a increase expression of E-cadherin. Besides, the mRNA expression of MMP-2/9, EMMPRIN, CD44 and E-cadherin was also diminished in PANC-1 cells treated with α-Solanine. These results indicated that α-Solanine exhibited anti-metastatic ability partly due to its inhibiting effect on the expression of MMP-2, MMP-9, EMMPRIN, CD44 and E-cadherin.

Angiogenesis is crucial for tumor initiation, development and metastasis. Secretion of mutiple angiogenic factors, including PDGF, bFGF,eNOS and VEGF which has been identified as the most important pro-angiogenic factor [41], is a initial step of angiogenesis. In our vitro study, α-Solanine markedly attenuated tube formation of HUVEC, which is one of initial events of angiogenesis. We further found that the expression of VEGF and eNOS were downregulated in PANC-1 cells after α-Solanine treatment. Therefore, we indicated that α-Solanine suppressed tumor angiogenesis partially via inhibition of VEGF and eNOS expression.

In vitro study, we also observed whether α-Solanine intervened in the Wnt/β-catenin, Akt/mTOR, Stat3 and NF-κB pathways which had a critical influence on tumor cells proliferation, angiogenesis and metastasis. The result demonstrated that α-Solanine significantly induced β-catenin phosphorylation while moderating the level of β-catenin and TCF-1 in nucleus. Furthermore, Akt, mTOR and Stat3 phosphorylation were alleviated by α-Solanine. NF-κB activity is closely associated with apoptosis inhibition, angiogenesis and metastasis. Our study verified that α-Solanine could abolish NF-κB protein level in the nucleus, suggesting that α-Solanine subdued the activity of NF-κB. Hence, the inhibitory effect of α-Solanine on Wnt/β-catenin, Akt/mTOR, Stat3 and NF-κB pathways may be involved in the anti-proliferation, anti-angiogenesis and anti-metastasis of α-Solanine.

For the possible clinical antitumor efficacy of α-Solanine, it is necessary that its antitumor efficacy in vitro should be translated in in vivo condition. Therefore, we evaluated whether α-Solanine exhibits similar effects in nude mice. In our vivo study, we found that α-Solanine significantly inhibited the growth of PANC-1 tumor xenografts with time at the end of 2 weeks. Proliferation, angiogenesis and metastasis are widely used biomarkers employed for diagnosis and measurement of aggressive of solid tumors [42], [43]. Hence, we examined pancreatic xenograft tumors by immunohistochemical staining for PCNA and VEGF and Western blot for MMP-2/9. PCNA is essential for DNA replication during S-phase of cell cycle and hence accelerate DNA synthesis and promote cellular proliferation [44]. PCNA protein level was increased when β-catenin accumulated in nucleus [45]. We found α-Solanine treated tumors showed reduced expression of MMP-2/9, especially MMP-9. Immnunohistochemical analysis revealed that the activity of PCNA was markedly decreased in α-Solanine treated group. In addition, a obviously decrease (70%) in staining for VEGF was found in α-Solanine treated group compared with control group. Consequently, these results showed that in vivo antitumor efficacy of α-Solanine in PANC-1 tumors is associated with an inhibition of metastasis, cell proliferation and tumor angiogenesis.

In summary, we demonstrated the antitumor efficacy of α-Solanine (nontoxic concentrations) on proliferation, migration, invasion, angiogenesis and metastasis of humanpancreatic cancer cells in vitro. Inhibition of Wnt/β-catenin, Akt/mTOR, Stat3 and NF-κB pathways was partially contributed to antitumor efficacy of α-Solanine. In vivo study, we also verified the anti-proliferation, anti-angiogenesis and anti-metastasis of α-Solanine on PANC-1 tumor xenografts.
